# Combination Treatment with Intravenous Tigecycline and Intraventricular and Intravenous Colistin in Postoperative Ventriculitis Caused by Multidrug-resistant Acinetobacter baumannii

**DOI:** 10.7759/cureus.3888

**Published:** 2019-01-15

**Authors:** Cezar J Mizrahi, Shmuel Benenson, Samuel Moscovici, Carlos Candanedo, Mony Benifla, Sergey Spektor

**Affiliations:** 1 Neurosurgery, Hadassah-Hebrew University Medical Center, Jerusalem, ISR; 2 Internal Medicine, Hadassah-Hebrew University Medical Center, Jerusalem, ISR

**Keywords:** carbapenem-resistant acinetobacter baumannii, colistin, tigecycline, multidrug-resistant acinetobacter baumannii, ventriculitis

## Abstract

Nosocomial infections with multidrug-resistant (MDR) pathogens are a life-threatening complication in neurosurgery. An MDR Acinetobacter baumannii (A. baumannii) central nervous system (CNS) infection following neurosurgery has been previously reported and was treated with relative success using intraventricular and/or intravenous (IV) colistin, IV tigecycline, or IV colistin-rifampicin combination therapy. We present a case of MDR A. baumannii in a 13-year-old girl following parietal craniotomy for the resection of a right intraventricular meningioma. Several days after surgery, the patient presented with clinical, radiological, laboratorial, and microbiological evidence of carbapenem-resistant A. baumannii ventriculitis. She was treated with IV colistin and then with combined intraventricular-IV colistin, with partial failure. The combined treatment of IV tigecycline and associated intraventricular and intravenous colistin was started and significant improvement was seen clinically and radiologically, with negative cultures after one week. To the best of our knowledge, this is the first case of a successful combination of intraventricular and IV colistin combined with IV tigecycline after a partial treatment failure with intraventricular and IV colistin alone.

## Introduction

Nosocomial infections with multidrug-resistant (MDR) pathogens are a life-threatening complication in neurosurgery. Acinetobacter baumannii is a gram-negative, aerobic, non-fermentative, oxidase-negative coccobacillus that may potentially cause respiratory infections, bacteremia, meningitis, and ventriculitis [1]. An MDR Acinetobacter baumannii central nervous system (CNS) infection following a neurosurgical procedure was previously reported [1-3], with relative treatment success after intraventricular and/or intravenous (IV) colistin [4-5], IV tigecycline [6-7], and IV colistin-rifampicin combination therapy [8].

We present a case of post-neurosurgical carbapenem-resistant A.baumannii ventriculitis treated with intraventricular and IV colistin in combination with IV tigecycline after a partial treatment failure of intraventricular and IV colistin.

## Case presentation

**Figure 1:**
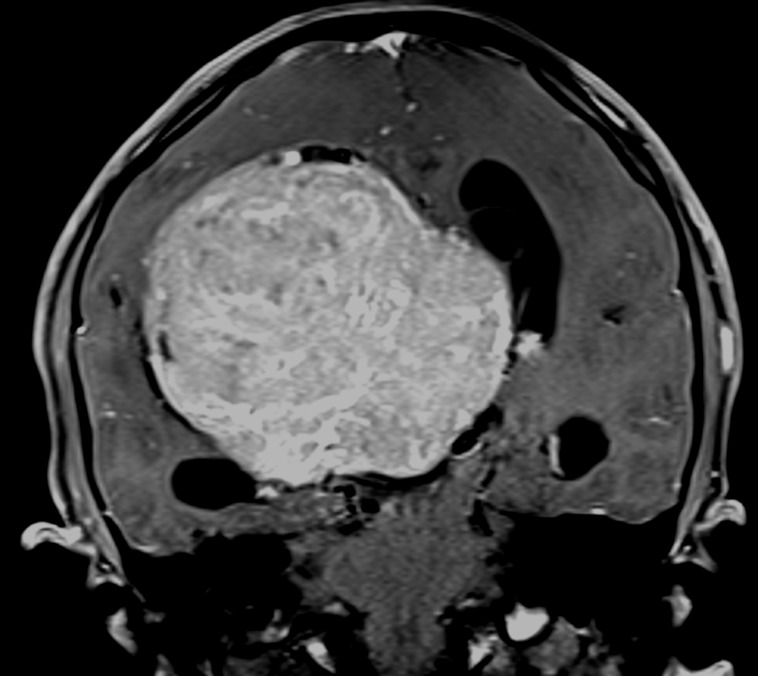
Coronal T1-weighted gadolinium-enhanced MRI in a 13-year-old female The patient was referred to our outpatient clinic due to a one-year history of headache. At histopathology, the large homogeneously enhancing intraventricular mass located in the right lateral ventricle, causing obstructive hydrocephalus, was found to be a transitional meningioma, WHO I MRI: magnetic resonance imaging: WHO: World Health Organization

A healthy 13-year-old female with an unremarkable medical history was referred to our outpatient clinic due to a one-year history of headache. Routine hematological tests and serum chemistry were normal. T1-weighted gadolinium-enhanced brain magnetic resonance imaging (MRI) (Figure 1) revealed a large, homogeneously enhancing intraventricular mass in the right lateral ventricle with associated obstructive hydrocephalus.

Gross total removal of the tumor was achieved. Surgery was uneventful and a right external ventricular drain (EVD) was placed after tumor resection. The histopathology of the tumor was a transitional meningioma, World Health Organization (WHO) I.

**Figure 2:**
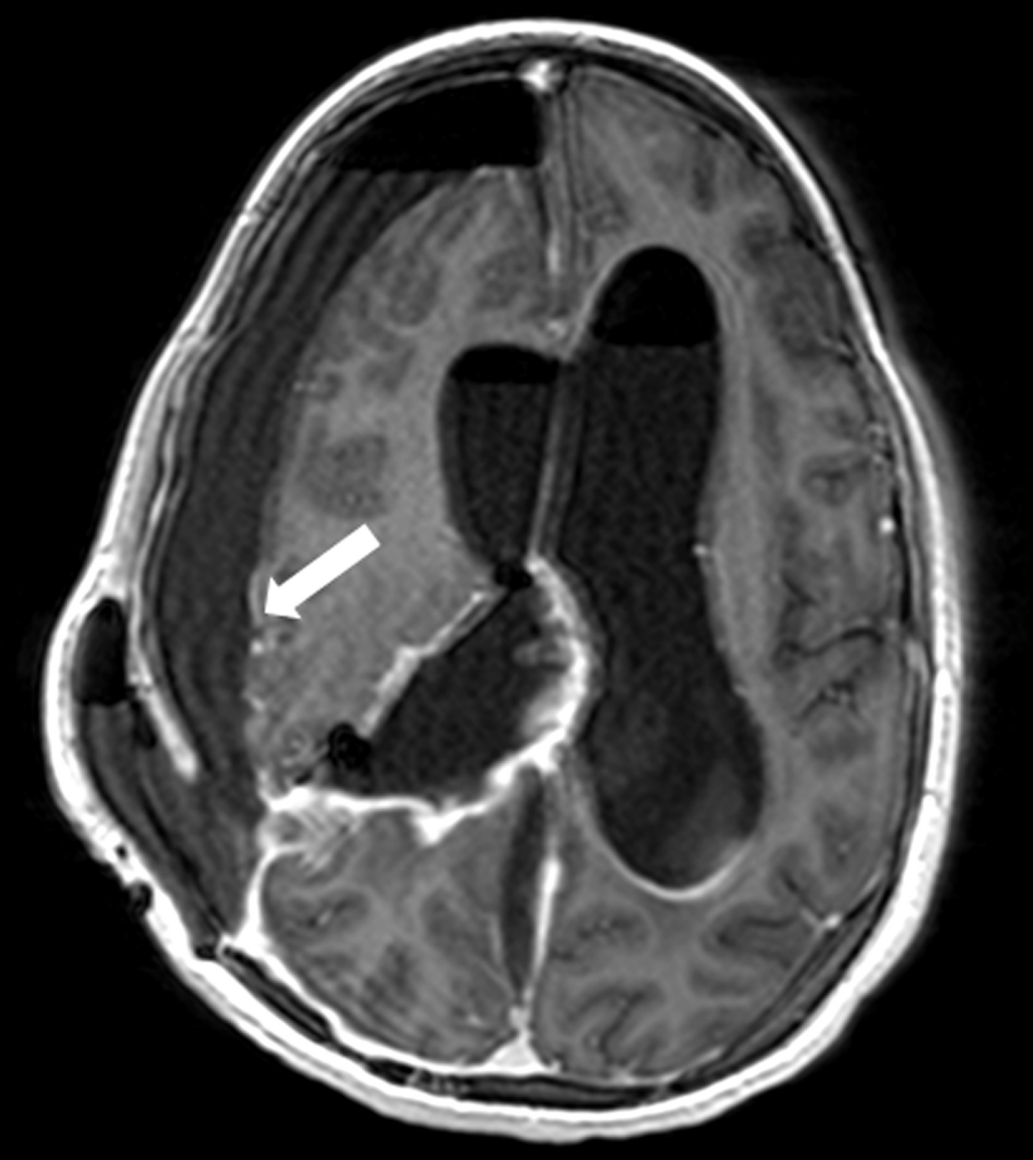
T1-weighted gadolinium-enhanced axial MRI Note the right subdural enhancing collection (arrow). MRI: magnetic resonance imaging

**Figure 3:**
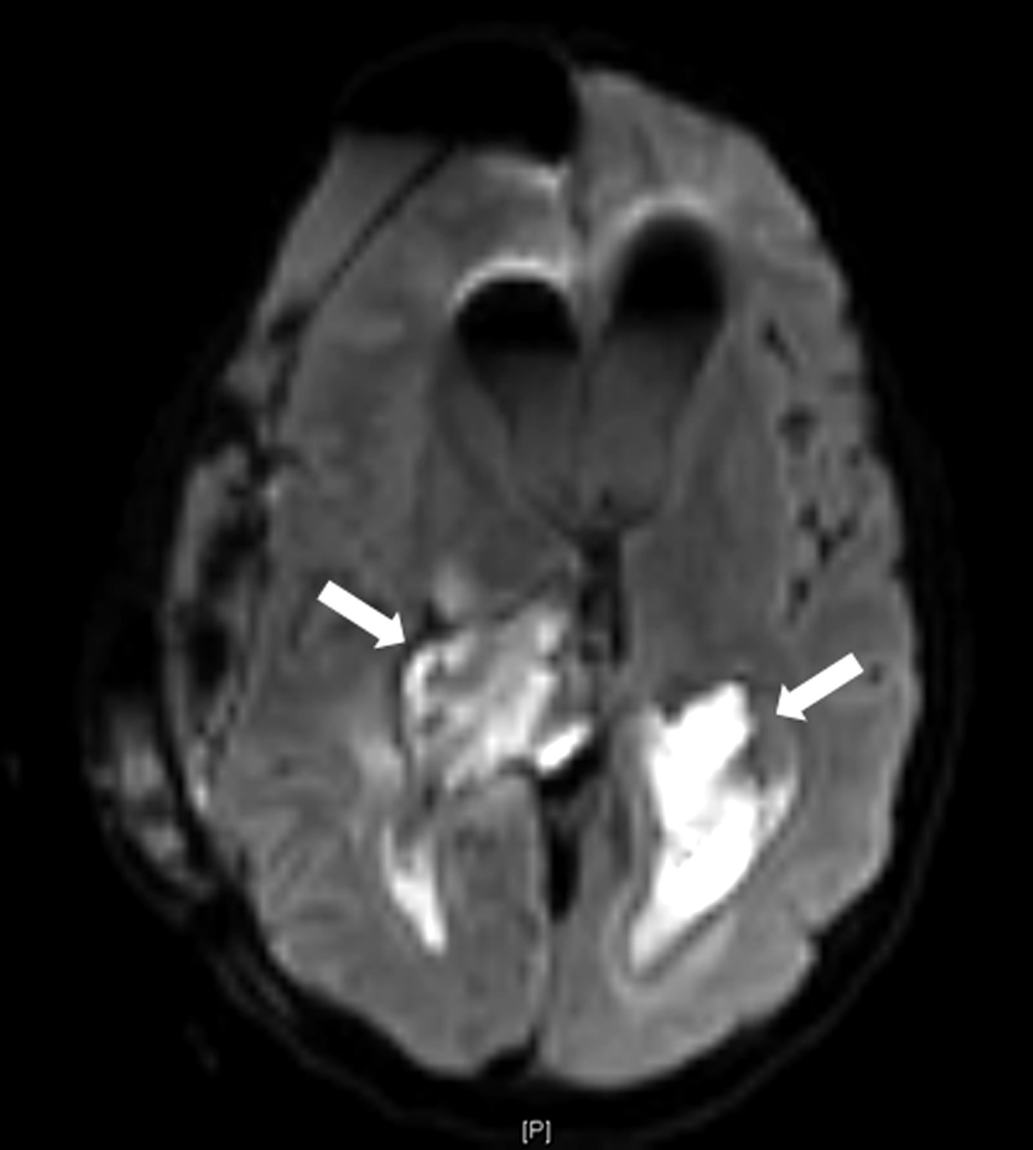
DWI MRI Note the diffuse intraventricular restriction on both lateral ventricles (arrows), characteristic of subdural empyema and severe ventriculitis. DWI: diffusion-weighted imaging; MRI: magnetic resonance imaging

The patient’s immediate postoperative recovery was marked by two episodes of wound cerebrospinal fluid (CSF) leak, which were treated conservatively with local stitches. However, five days after surgery, she developed a high fever (39.8°C) and a purulent discharge from the EVD. The physical examination revealed neck stiffness. She had marked leukocytosis (38.5×109/l) although her biochemical parameters were within normal limits. The CSF examination obtained via the EVD revealed severe hypoglycorrhachia (2.2 mg/dL versus an expected level of 66–77 mg/dL at blood glucose level 111 mg/dL), with a hyper proteinorachie of 2581 mg/L (normal <450 mg/L). After sending CSF, urine, and blood samples for cultures, IV vancomycin (2g/day) and ceftazidime (6g/day) were initiated empirically. On the following day, the CSF culture was positive for carbapenem-resistant A. baumannii and the antibiotic protocol was changed to the maximum recommended colistin dose according to patient weight (6 million units/day). After three days, the patient’s high fever (40.1°C), neck stiffness, and leukocytosis (33.7×109/l) persisted, with worsening hypoglycorrhachia (0.44 mg/dL versus an expected level of 58–68 mg/dL at blood glucose level 99 mg/dL) and hyper proteinorachie (6967 mg/L). The CSF culture remained positive for carbapenem-resistant A. baumannii. T1-weighted gadolinium-enhanced (Figure 2) and diffusion-weighted imaging (DWI) MRI studies (Figure 3) revealed a right subdural enhancing collection with a diffuse bilateral intraventricular restriction, subdural empyema, and severe ventriculitis.

The craniotomy site was reopened, the subdural empyema was drained, and the EVD was replaced by a new catheter, without surgical complications. Intraventricular colistin (150,000 units (10 mg) once daily) was added to the antibiotic treatment after surgery. After five days, fever (38.1°C) and neck stiffness were somewhat reduced and the leukocytosis was improved (18.1×109/l); however, hypoglycorrhachia persisted (11 mg/dL versus an expected level of 52–61 mg/dL at blood glucose level 89 mg/dL), hyper proteinorachie had deteriorated (9433 mg/L), and CSF culture for carbapenem-resistant A. baumannii remained positive. The isolate was found to be sensitive to tigecycline (minimum inhibitory concentration (MIC) of 0.38 µg/ml), and IV tigecycline was added to the combination therapy (50 mg twice daily, following a loading dose of 100 mg). After one week, the patient showed complete clinical recovery, resolution of the leukocytosis, and sterile CSF; however, hyper proteinorachie persisted (1996 mg/L). The EVD was removed two weeks after the second surgery and intraventricular colistin was discontinued. IV tigecycline-colistin continued for four more weeks. The patient developed headaches and blurred vision. Contrast-enhanced head CT revealed an improvement of the intraventricular enhancement and subdural collection but an enlargement of the ventricular system and transependymal edema with a communicating hydrocephalus. The patient underwent ventriculoperitoneal (VP) shunt insertion. There were no complications or signs of further VP shunt infection or hydrocephalus. She was discharged at the end of therapy, six weeks after the reopening of the craniotomy site, without any evidence of infection. At the six, 12, 18, and 24-month follow-up, the patient had reached a Glasgow Outcome Score of 5 with no sign of a new infection.

## Discussion

We present a case of ventriculitis caused by MDR A. baumannii, which was successfully treated with a combination of intraventricular and IV colistin and IV tigecycline, in a 13-year-old patient. To the best our knowledge, this is the first report of a successful combination of colistin and tigecycline, after the partial failure of IV and intraventricular colistin treatment in a postoperative neurosurgery patient.

CNS infections caused by MDR A. baumannii present a clinical challenge in neurosurgery patients. The prevalence of postoperative infections caused by carbapenem-resistant A. baumannii has continuously increased in recent years. Successful treatment with intrathecal colistin was pioneered in 1999 in two patients, a 16-year old boy who underwent craniotomy and external ventricular drain insertion for the treatment of a fourth ventricle hemangioblastoma and a 34-year-old woman who required EVD placement due to a spontaneous subarachnoid hemorrhage complicated with hydrocephalus. Since then, success treating a carbapenem-resistant A. baumannii CNS infection after neurosurgical procedures with intrathecal or intraventricular colistin has been reported [5].

Structurally related to minocycline, tigecycline was the first drug in the glycylcycline antibiotic class. Alterations to the molecule resulted in an extended-spectrum antibiotic, exhibiting activity against a broad range of MDR pathogens, including carbapenem-resistant A. baumannii strains [9]. Despite its high efficacy against MDR pathogens, tigecycline showed only modest penetration into the cerebrospinal fluid (CSF) in healthy volunteers [10], although CSF penetration may be facilitated by inflamed meninges. Recently, studies demonstrated the successful treatment of carbapenem-resistant A. baumannii meningitis with IV tigecycline [6].

Motaouakkil et al. [8] described the first clinical report of colistin combined with rifampicin for the treatment of an MDR A. baumannii infection in 26 critically ill patients, including 16 cases of nosocomial pneumonia and nine cases of bacteremia but only one case of meningitis. The patient with meningitis was treated with intrathecal colistin and IV rifampicin.

## Conclusions

Treatment combining IV tigecycline with intraventricular and IV colistin may be a useful tool against carbapenem-resistant Acinetobacter baumannii ventriculitis when colistin treatment alone has failed.
